# Calculated initial parenteral treatment of bacterial infections: Pharmacokinetics and pharmacodynamics

**DOI:** 10.3205/id000061

**Published:** 2020-03-26

**Authors:** Hartmut Derendorf, Tobias Heinrichs, Tobias Reimers, Cordula Lebert, Alexander Brinkmann

**Affiliations:** 1 Department of Pharmaceutics, College of Pharmacy, University of Florida, Gainesville, USA; 2 Bayer AG, Klinische Pharmazie, Leverkusen, Germany; 3 Apotheke, Klinikum Nürnberg, Nuremberg, Germany; 4 Klinik für Anästhesie, operative Intensivmedizin und spezielle Schmerztherapie, Klinikum Heidenheim, Germany

## Abstract

This is the third chapter of the guideline “Calculated initial parenteral treatment of bacterial infections in adults – update 2018” in the 2^nd^ updated version. The German guideline by the Paul-Ehrlich-Gesellschaft für Chemotherapie e.V. (PEG) has been translated to address an international audience.

The chapter features the pharmacokinetic and pharmacodynamics properties of the most frequently used antiinfective agents.

## Zusammenfassung

Dies ist das dritte Kapitel der von der Paul-Ehrlich-Gesellschaft für Chemotherapie e.V. (PEG) herausgegebenen S2k Leitlinie „Kalkulierte parenterale Initialtherapie bakterieller Erkrankungen bei Erwachsenen – Update 2018“ in der 2. aktualisierten Fassung.

Dieses Kapitel bespricht die pharmakokinetischen und pharmakodynamischen Eigenschaften der am häufigsten eingesetzten Antiinfektiva.

## Pharmacology

In addition to the antimicrobial properties (pharmacodynamics) of a substance, the pharmacokinetic properties, i.e. the behavior in an organism, play a decisive role. Ultimately the question is whether the concentrations at the site of action are sufficient to inhibit the pathogens, kill them and possibly prevent the development of resistant pathogens. Adverse drug reactions and interactions should be minimized.

For the purpose of predicting efficacy, one speaks of PK/PD (pharmacokinetics/pharmacodynamics) when pharmacokinetic parameters or, in the simplest case, plasma and tissue concentrations are associated with the antimicrobial properties in vitro or in vivo.

## Pharmacokinetics

Pharmacokinetic properties of drugs are determined by their physicochemical characteristics. The acid or base strength of a substance, its lipophilicity or hydrophilicity determine how the substance behaves under the physiological conditions of an organism. For example beta-lactam antibiotics and aminoglycosides are poor at penetrating membranes and therefore are located mainly in the extracellular space. An overview of pharmacokinetic parameters of individual substance groups is shown in Table 1 (Tab. 1).

An important pharmacokinetic parameter that describes the distribution of the drug in the body is the volume of distribution. Lipophilic substances, which can easily pass through membranes, are passively taken up intracellularly. Their volume of distribution is therefore high; with fluoroquinolones and macrolides it can be a multiple of the body volume. Substances with large volumes of distribution have lower plasma and interstitial levels but high intracellular concentrations. Water-soluble substances, on the other hand, penetrate cell membranes with difficulty and therefore mainly remain in the plasma and interstitium. Most pathogens are located in the interstitium, so concentration in these cases is crucial.

An important aspect of drug distribution is protein binding in serum. Depending on their physicochemical properties, antibiotics mainly bind to albumin. 

Concentration-dependent binding is reversible. There is a dynamic balance between the free and the bound portion. In general, only the free, non protein-bound portion of an antibiotic is responsible for its action. As demonstrated for some antibiotics, high protein binding need not adversely affect the efficacy of a substance as long as there are sufficiently high unbound concentrations at the site of action. Clinical studies that appear to demonstrate a negative influence of protein binding were often performed with low total doses [1], [2], [3]. Furthermore, protein binding plays a role in kidney replacement procedures. Only the free, non protein-bound active substance portion can be eliminated via the artificial membranes of a kidney replacement procedure.

Equally significant for predicting efficacy is the question of tissue concentration. Tissue concentrations, as determined from biopsy material or surgical resectates, represent average concentrations in tissue homogenate. They do not adequately represent the complex processes or the heterogeneous distribution in the tissue. The measurements of tissue concentrations are important, for example when comparing two substances or substance groups.

Big progress was made in this area with the development of microdialysis. The measurement of antibiotic concentrations in compartments such as cerebrospinal fluid, alveolar film, pleural fluid, peritoneal fluid, pancreatic and prostatic fluid is important. Disease-related microcirculatory disturbances with compromised tissue perfusion, cell membranes with special anatomic structures and the presence of specific tissue receptors can be obstacles to the even distribution of antibiotics and thus influence treatment success. Table 2 (Tab. 2) shows the accessibility of different compartments for antibiotics. Thus, not only the physicochemical properties of the anti-infective agents but also the perfusion of the deep compartments play a crucial role in the actual site concentration [4], [5], [6].

## Interaction between pharmacokinetics and pharmacodynamics

Since insufficient data is available on the concentration profiles at the site of infection, the pharmacokinetic evaluation of the various substances is usually carried out today using the different plasma concentrations; in severely ill intensive care patients, site concentrations may differ from the measurements in the primary compartment (serum, plasma) (especially in infections in deep compartments: lungs, bones, soft tissues) [4], [6]. Depending on the mechanism of action, different indices are recommended for the different groups of active ingredients to manage treatment.

The differences in the pharmacodynamic profile of the antibiotic groups are also explained by their different modes of action – concentration-dependent effect of fluoroquinolones, aminoglycosides, tetracyclines and glycylcyclines (tigecycline) and the time-dependent (non concentration-dependent) effect of beta-lactam antibiotics, lincosamides and macrolides (Table 3 (Tab. 3)). In the case of aminoglycosides, fluoroquinolones and cyclic lipopeptides (daptomycin), it has been shown that the ratio of peak concentration (C_max_) to the minimal inhibitory concentration (MIC) of the pathogen correlates with treatment success. For beta-lactam antibiotics, on the other hand, it is the percentage of the dosing interval in which the plasma concentration is above the pathogen MIC (t>MIC or %t>MIC). For fluoroquinolones and cyclic lipopeptides (daptomycin), the quotient of AUC (area under the curve) and MIC are considered predictive (the area under the 24-hour concentration time curve relative to the MIC: AUC_24_/MIC). This also applies to the group of glycopeptides. Previous findings on oxazolidinones (linezolid, tedizolid) indicate that both concentration and duration of exposure are relevant. The validation of these models for humans has been shown for some antibiotic groups.

In particular, in immunosuppressed patients and in infections in hard to reach compartments (abscesses, osteomyelitis, meningitis, necrotizing infections, see also Table 2 (Tab. 2)), the consideration of PK/PD indices in the choice of dosage regimen is of crucial importance. Also, the pharmacokinetic characteristics in the critically ill – which are affected by their hyperdynamic circulatory situation, endothelial damage, increased capillary permeability, hypoalbuminemia, extracorporeal circuits, intravenous administration of large amounts of fluid or administration of vasopressors – can contribute to an increased volume of distribution and by increasing renal perfusion in the absence of relevant organ dysfunction to an increased clearance of hydrophilic antibiotics and reduction of their plasma concentration [4], [6]. For such seriously ill patients, other PD indices may be important besides MIC. For treatment success in certain groups of pathogens (non-fermenters, e.g. *Pseudomonas aeruginosa*) including the avoidance of resistance development, concentration profiles may be more favorable in which the site concentrations remain well above the MIC (corresponds to MPC, mutant prevention concentration) [4], [7]. 

The data on PK/PD correlations offer the possibility of adjusting the dosage individually using therapeutic drug monitoring (TDM), especially in high-risk populations (such as critically ill patients, geriatric patients, patients with organ failure, infections with multidrug-resistant pathogens [e.g. extended-spectrum beta-lactamase (ESBL) producers]) [6], [8], [9], [10].

Clearance and volume of distribution determine the half-life of a substance. These parameters also co-determine the time the plasma concentration is above the MIC and for the total exposure (AUC) and play an important role in the calculation of the dosing interval.

Decreased function of the drug-eliminating organs (especially the kidneys and the liver) results in reduced clearance of antibiotics and prolongs the half-life, which may be one reason for the increased rate of adverse effects. The relevance of impaired renal and hepatic function plays a lesser role for antibiotics with a wide therapeutic range (broad concentration range between the effective and the toxic levels, for instance for penicillins, cephalosporins, carbapenems, macrolides, lincosamides, fluoroquinolones, linezolid) than for antibiotics with a narrow therapeutic range (such as aminoglycosides or vancomycin). Nevertheless, in intensive care patients it has been shown that increased plasma levels of beta-lactam antibiotics are associated with a poorer neurological outcome [5]. In addition to the microbiological efficacy, the extent of renal and extrarenal elimination as well as any potential nephro- and/or hepatotoxic potential of the antibiotics themselves or their metabolites play an important role in the selection of suitable antibiotics. These antibiotics (potentially nephrotoxic: aminoglycosides, vancomycin, teicoplanin, telavancin; potentially hepatotoxic: amoxicillin/clavulanic acid, flucloxacillin, fluoroquinolones, tetracyclines, rifampicin) should only be administered in life-threatening situations if the corresponding organ has impaired function. Possible risks due to the accumulation of potential toxic metabolites in patients with pronounced renal and hepatic insufficiency should also be considered. In principle, antibiotics with high extrarenal elimination should be selected in cases of impaired renal function and, in hepatic insufficiency, antibiotics with a predominantly renal excretion mode.

Antibiotics which are predominantly eliminated via the kidneys to varying degrees are also secreted by glomerular or tubular filtration (e.g. penicillins) or reabsorbed. If renal function is impaired, the dosage should be adjusted to the degree of renal impairment according to creatinine clearance. The following are crucial in determining the need for dose adjustment:

- the proportion of renal elimination of the drug in normal renal function,
- the toxicity of the substance,
- the degree of renal impairment; and
- the increase of creatinine clearance beyond normal levels (for example with reduced muscle mass, pregnancy or early stage diabetes mellitus).

In general the dosage specifications of the manufacturers should be followed. If these are not available, the dosing regimen for renal insufficiency should be adjusted by calculating the individual elimination fraction (Q) according to Dettli [11], [12].

Helpful links for dose adjustment in renal insufficiency:

- http://www.infektio.de/antiinfektiva/dosierung-bei-niereninsuffizienz/
- http://www.dosing.de/

Critically ill intensive care patients have a special status in terms of substance-specific pharmacokinetics. Recommended dosages and sensitivities (tested as sensitive, intermediate or resistant) are based on the assumption that the pharmacokinetics of the drug are equivalent to those of a “standard patient”. In fact, however, the distribution and elimination capacity of drugs in the critically ill is very variable and difficult to predict. The renal function of patients with severe infections alone shows great inter- and intra-individual variability, so that the drug clearance and thus the optimal dosage of predominantly renally excreted anti-infective drugs can vary by a factor of 10 [13]. This problem is not only clinically apparent with beta-lactam antibiotics [13] but also with reserve substances such as linezolid [14]. A review article provides helpful guidance on individualized dosing of anti-infective agents (e.g. web-based calculation programs such as CADDy [Calculator to Approximate Drug Dosing in Dialysis] in seriously ill intensive care patients [6]. Patients with organ replacement procedures (such as renal replacement procedures [hemodialysis, hemofiltration] [6], [8], [15], ECMO [16], ECLS) present a particular challenge here.

Unlike creatinine clearance in renal insufficiency, clinical scores in hepatic insufficiency (Child-Pugh score, MELD score) are not good predictors of drug metabolization and elimination.

Liver diseases have a different, unpredictable influence on the individual cytochrome P450 isoenzymes. Existing tests allow only a rough assessment of the function of the individual isoenzymes. The reduction in hepatic clearance and the associated need for dose adjustment may be relevant to antibiotics that are almost exclusively metabolized by liver enzymes, predominantly those with high lipophilicity and low polarity that can be poorly eliminated via the kidney (antibiotics with high extrarenal clearance: clindamycin, tedizolid, chloramphenicol and minocycline). When dosing with other tetracyclines, clavulanic acid, flucloxacillin, macrolides or streptogramins, higher grade hepatic insufficiency with reduced metabolization performance must also be considered. For antibiotics with a high presystemic elimination rate (e.g. ciprofloxacin), hepatic impairment may significantly increase the bioavailability after oral administration and thus the plasma concentration.

For all stages of renal and hepatic insufficiency, the loading dose (initial dose), which depends on the volume of distribution, should be identical to that for a healthy kidney or liver. Otherwise, initially reduced doses of antibiotics may take several days to reach an effective level. Since the success of antibiotic treatment mainly depends on the initial selection and an adequate dosage, this would jeopardize treatment success.

The dosage of antibiotics in overweight patients is a particular pharmacotherapeutic problem. The kinetics of many antibiotics are sometimes unpredictable due to unusual distributional processes in these patients. There is no clear relationship between the lipophilicity of the substances and their distribution in obese patients. Altered volume of distribution, clearance and problems in assessing kidney function using creatinine clearance are just some of the reasons that often cause overweight patients to be inadequately treated with standard doses of antibiotics. Subtherapeutic concentrations may then lead to clinical treatment failure and development of resistance, while supratherapeutic/excessively high concentrations usually lead to undesirable side effects (with the exception of aminoglycosides). Since an increased volume of distribution and increased clearance is generally to be expected in these patients, a weight-adapted dose adjustment is necessary. Which weight (TBW – total body weight, IBW – ideal body weight, LBW – lean body weight or ABW – adjusted body weight) should be used as the basis for the dose calculation is dependent both on the antibiotic itself (e.g. in tigecycline with a distribution volume of 7 to 10 l/kg [17]) as well as the type and duration of administration [18], [19], [20], [21].

Hydrophilic antibiotics (beta-lactams, aminoglycosides, glycopeptides) [22] are less well distributed in adipose tissue. When dosing these antibiotics IBW or ABW are usually used [23]. Using TBW can lead to overdoses. In contrast, lipophilic antibiotics (fluoroquinolones, macrolides, clindamycin, tetracyclines, tigecycline, cotrimoxazole, rifampicin, chloramphenicol) [22] have a higher volume of distribution. Consequently, increased adipose tissue in obese patients also leads to an increase in the volume of distribution compared to patients with normal weight. TBW tends to be used for dosing in this case [23]. It should be noted that the degree of hydrophilicity or lipophilicity within the two groups (hydrophilic and lipophilic antibiotics) differs from antibiotic to antibiotic.

For special patient populations (those with CF, sepsis, neutropenia, burns, or high body weight) Therapeutic Drug Monitoring (TDM) is recommended [6], [8], [9], [10] but only a few antibiotics have rapid tests available (for example, aminoglycosides, glycopeptides). Special dosage guidelines must be observed in the aforementioned patient groups. The different pharmacokinetic characteristics of the individual substances are summarized in Table 1 (Tab. 1).

## Therapeutic drug monitoring

Many antibiotics are characterized by significant inter- and intra-individual differences in pharmacokinetic properties, especially in elimination behavior and volume of distribution. This is especially true in intensive care patients with severe sepsis, septic shock and consecutive multiple organ failure and profound changes in distribution spaces (e.g. capillary leak and infusion treatments) [6], [8]. As a result, even standard doses can result in a wide range of plasma concentrations [13], which, on the one hand, threatens the risk of under-dosing with insufficient therapeutic effect, on the other hand excessive plasma levels with the risk of undesirable toxic effects. The aim of therapeutic drug monitoring (TDM) is to find the optimal individual dosage for the patient taking into account pharmacokinetic principles and measurements of the drug concentration in the patient’s blood [6], [7], [8].

Prerequisites or indications for conducting TDM above all are:

- Therapeutic and toxic effects are in a concentration related cause and effect relationship.
- The substance has a narrow therapeutic range and exceeding the concentration range by even relatively degree can lead to toxic effects.
- The pharmacokinetics of the drug are subject to significant intra- and inter-individual variability, especially in intensive care patients with severe sepsis and septic shock.
- Pharmacokinetic target parameters (C_max_, C_min_, AUC) are known.
- Sufficiently sensitive methods for determining concentration are available involving a reasonable amount of effort.

For many antibiotics, e.g. penicillins and cephalosporins, the risk of unwanted toxic effects is rather low, since they have a relatively large therapeutic range. For these antibiotics, treatment based on blood level is only recommended for certain patient groups (e.g. intensive care patients) [4], [6], [8], [10], [13]. In a mixed intensive care group, dosage adjustment is necessary in 20–30% of cases [4], [8]. Intensive care patients with elevated creatinine clearance are at particular risk of the associated under-dosing [4], [6], [8], [24], [25]. The measurement of beta-lactam concentrations is currently not widespread, as dedicated PK/PD targets and dose-adjustment strategies are currently being debated in scientific circles [6], [10]. The measurement is predominantly done by chromatography. Commercial measuring methods are not available in Germany [6], [8], [10]. Drugs where TDM is strongly recommended for their safe use include aminoglycosides and glycopeptides. Table 4 (Tab. 4) gives recommendations on the target ranges for the peak and trough levels of the most commonly used aminoglycosides and glycopeptides taking into account different patient populations.

In treatment with aminoglycosides, single administration of the total daily dose has become more widespread, with increased clinical effectiveness, lower toxicity and economic advantages [26], [27], [28], [29], [30], [31], [32], [33], [34], [35], [36]. Taking into account accepted PK/PD parameters, aminoglycoside peak levels well above the MIC of the pathogen (C_max_/MIC>10) are aimed for [37], [38]. The mean MIC of gentamicin is 2 mg/l for pathogens with reduced sensitivity (e.g. for *Pseudomonas aeruginosa*); thus, peak levels of at least 20 mg/l should be aimed for [39]. 

In the treatment of endocarditis and neutropenic patients, single dose administration is sufficient in most cases. For severe endocarditis (enterococci, heart valve prostheses), single dose administration is not recommended and multiple administration recommended, for example in combination with a synergistic antibiotic attacking the cell wall [40].

In treatment with the glycopeptide antibiotics vancomycin and teicoplanin the aims are permanent concentrations above the MIC of the relevant pathogens in accordance with their pharmacodynamic parameters. As mandated by TDM, trough levels are monitored [41]. For the treatment of life-threatening infections (meningitis and pneumonia) and reduced sensitivity agents, vancomycin trough levels of 15–20 mg/l should be the target [42], [43], [44]. However, the increased risk of nephrotoxicity above a vancomycin trough level >15 mg/l should be taken into account [45]. Evidence from recent literature suggests that continuous administration of vancomycin reduces the likelihood of nephrotoxic side effects [46], [47], [48], [49]. 

For the treatment of bone or prosthetic infections, teicoplanin trough levels of 20–25 mg/l are recommended [50]. When teicoplanin is used to treat bacterial endocarditis, trough levels should be at least 30–40 mg/l [51]. Trough levels above 60 mg/l are considered toxic [52].

## Continuous or prolonged infusions of beta-lactam antibiotics

Beta-lactam antibiotics are effective if the MIC of the pathogens is exceeded as permanently as possible during the growth phase of the cell wall. Initially, the bactericidal activity increases with increasing concentrations of the antibiotic up to 4 to 5 times the MIC but higher levels will not improve the therapeutic outcome. This pharmacokinetic-pharmacodynamic relationship is described as a time-dependent (non-concentration-dependent) bactericide. For beta-lactam antibiotics, the concentration of unbound antibiotic should exceed the MIC of the pathogen at the site of infection for at least 40–60% of that time [53], with approximately 40% for carbapenems and higher for cephalosporins; penicillins are in between. These data are derived from animal studies. Clinical trial results in intensive care patients suggest that keeping above the MIC 100% of the time can improve outcome [54], [55], [56], [57], [58]. Since in intensive care patients with severe infections in deep compartments the plasma concentrations measured in the context of TDM do not correspond to active site concentrations, as a PK/PD target some experts recommend keeping the plasma level 4 to 5 times above the MIC for the full dosing interval [4], [6], [8], [10]. 

The pharmacokinetic properties of beta-lactam-antibiotics do not show great variability one from another. Beta-lactam antibiotics are rapidly distributed in the extracellular space after parenteral administration. At steady state, similar concentrations are reached after intermittent administration and bolus administration followed by continuous infusion [59], [60], [61], [62], [63], [64].

The manufacturer’s dosage recommendations usually call for administering beta-lactam antibiotics 2 to 4 times (1 to 6 times) depending on the pharmacokinetic parameters. As a result, approved indications which are confirmed by clinical studies usually result in sufficient free efficacy levels which exceed the MIC of sensitive pathogens. However, intermittent application often fails to achieve the goal of exceeding the pathogen’s MIC permanently at the site of the infection, as shown in PK/PD simulations, experimental and clinical studies [4], [6], [8], [10]. This is especially true in patients with high extracellular distribution spaces and an increased clearance rate. In particular this includes patients with a hyperdynamic circulatory situation and a capillary leak, e.g. in sepsis, patients with cystic fibrosis, drainage, bleeding, large burns, ascites, severe pancreatitis, patients with a BMI >30 kg/m^2^, congestive heart failure, edema, haemofiltration (depending on balance), dialysis patients (pre-dialysis) and pregnant women [4], [6], [8]. In contrast, dehydrated patients, dialysis patients following dialysis and patients under volume restrictions have a lower volume of distribution than normal patients. For high-risk patients and in geriatrics, bespoke antibiotic treatment is therefore required [4], [6], [8], [65], [66], [67], [68], [69], [70], [71], [72], [73], [74], [75], [76], [77].

Recommendations for prolonged administration (over 3–4 hours) or continuous administration of beta-lactam antibiotics are based on theoretical considerations supported by experimental studies or simulations. Clinical examinations show advantages for prolonged or continuous administration with longer-lasting serum levels above the MIC even at lower daily doses [78], [79], [80], [81], [82], [83], [84], [85], [86],[87], [88], [89], [90], [91], [92], [93], [94], [95], [96] with comparable effectiveness and safety [71], [97], [98] in terms of clinical and microbiological efficacy. Advantages of prolonged antibiotic administration have been shown, above all, in severely ill intensive care patients (APACHE II score >17) [99]. There is currently no agreement regarding the superiority of continuous or intermittent administration [100], [101], [102], [103], [104]. A recent clinical study was not able to objectify differences in mortality [105]. However, another recent study underlined an improved healing rate following continuous administration [106]. This result was confirmed again by a recent meta-analysis [107]. Continuous application of beta-lactam antibiotics without TDM is not recommended without restriction because there is a risk of permanently falling below the pathogen’s MIC. Not only does falling below the MIC result in a lack of efficacy of the antibiotic but it can also favor the selection of resistant mutants. Securely reaching rational PK/PD goals can only be ensured with TDM and it is therefore of crucial importance in continuous application. Against this background prolonged application is much safer.

Beta-lactam antibiotics have limited stability after preparation. It is not only the degree of degradation that is crucial but also the type of decomposition products that have allergenic potential. This fact is insufficiently considered in numerous studies on the stability of the substances. According to these studies, solutions of beta-lactam antibiotics are considered to be stable over a period of time if their degradation level is below 10%. The extent of the degradation depends on the solvent, the effects of light, the concentration of the antibiotic, the type of application aids as well as their production and temperature. In the case of close-fitting pump application in outpatient parenteral antibiotic therapy (OPAT), significant stability losses must be taken into account due to increased ambient heat.

The use of the recommended solvents is of great practical importance to ensure optimum solubility and stability. Almost without exception, all penicillins (dry substances) must be dissolved in aqua ad injectabilia in order to accelerate the dissolution behavior and to ensure particle freedom. Further dilution is then usually possible in conventional infusion solutions. Many beta-lactam antibiotics show a number of incompatibility reactions with other medicines when administered in the same infusion system. The manufacturer’s information on compatibility must be observed.

The most common adverse drug reactions of penicillins are allergies and pseudoallergic reactions. The cause of these reactions is the presence of an unstable beta-lactam structure or specific side chains. Penicillins in solution vary in stability depending on their side chains and the pH. The degradation products of penicillins act as haptens and can form covalent bonds with the body’s own proteins. They form a hapten-protein complex that can induce an allergy-producing immune response. The degradation products of penicillins have a significant potential for allergic reactions. Further information on safety can be found in chapter 4 [108].

The nature and extent of the degradation of the beta-lactam antibiotics are substance-dependent. Acylaminopenicillins, isoxazolylpenicillins, cephalosporins and aztreonam are generally more stable than benzylpenicillin because of their structure. Ring-opening, however, is also possible with cephalosporins by nucleophilic or (more rarely) electrophilic attack, as the example of ceftazidime and other cephalosporins shows [109]. The chemical stability of carbapenems varies widely and above all depends on the concentration of the solution and the temperature [110], [111]. There are very contradictory data for the stability of various beta-lactam antibiotics in infusion solutions. Here the recommendation of the manufacturer of the product should be observed.

Like beta-lactam antibiotics in patients with severe infections, linezolid has a high variability in serum concentrations, with insufficient blood levels under standard dosage regimens. Current data indicate that a continuous application can also make a useful contribution to achieving PK/PD goals [112], [113].

### Conclusion

- Due to pharmacokinetic/pharmacodynamic considerations, prolonged or continuous infusion of beta-lactam antibiotics is superior to intermittent administration with respect to the therapeutic goal of exceeding the MIC of the pathogens as continually as possible.
- There is some clinical data on the significant superiority of this treatment regimen.
- Continuous and intermittent infusions of a beta-lactam antibiotic show a comparable side effect profile.
- Prolonged/continuous administration is recommended in patients whose pharmacokinetic parameters (volume of distribution, clearance) are significantly different from normal population data (for example, patients with sepsis and septic shock, cystic fibrosis, or patients with severe infections due to pathogens with reduced sensitivity). Continuous application is only recommended under TDM. Prolonged administration of a beta-lactam antibiotic is safe even without TDM.
- Prolonged/continuous administration of the antibiotic should always be preceded by a bolus dose.
- For substances with a high volume of distribution (for example tigecycline), a higher dose should be given initially.
- Possible economic advantages result from continuous administration, since similar serum concentrations in steady state compared to intermittent administration can be achieved with lower daily doses in patients who are not seriously ill.
- Some beta-lactam antibiotics are not suitable for continuous administration due to their low stability at room temperature. In these cases, only a prolonged infusion period (3–4 hours) is possible.
- The manufacturer’s recommendations regarding the type of solvents and the concentrations of the antibiotic solutions must be strictly adhered to. Deviations can result in considerably limited stability.
- Continuous administration of beta-lactam antibiotics requires separate access or lumen, as there are numerous incompatibility reactions with other medicinal products.
- Bioavailability data of the antibiotics for sequential therapy can be found in Table 5 (Tab. 5).

## Drug interactions

An important cause of unwanted side effects may be interactions with other drugs. In particular, the inhibition of hepatic monooxygenases, the cytochrome P450 enzymes, usually cause a higher risk of side effects, for example through some macrolides and fluoroquinolones as well as azole antifungals.

Also, induction-enhanced expression of enzymes of the cytochrome P450 enzyme system is possible, for example through rifampicin, barbiturates and carbamazepine. The consequence is a reduced plasma level with reduced effectiveness of the particular drug concerned.

More important examples of interactions of antibiotics with other drugs are presented in Table 6 (Tab. 6).

## Note

This is the third chapter of the guideline “Calculated initial parenteral treatment of bacterial infections in adults – update 2018” in the 2^nd^ updated version. The German guideline by the Paul-Ehrlich-Gesellschaft für Chemotherapie e.V. (PEG) has been translated to address an international audience.

## Competing interests

The authors declare that they have no competing interests.

## Figures and Tables

**Table 1:**
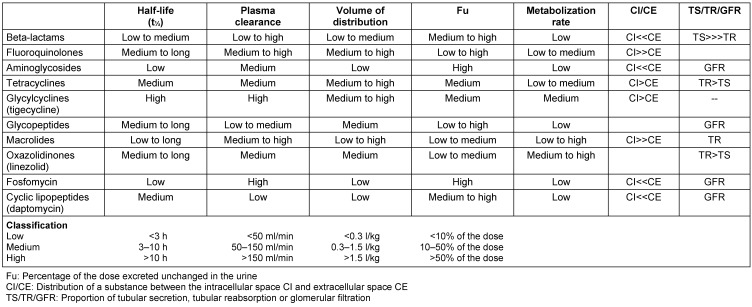
Pharmacokinetic characteristics of parenteral antibiotics

**Table 2:**
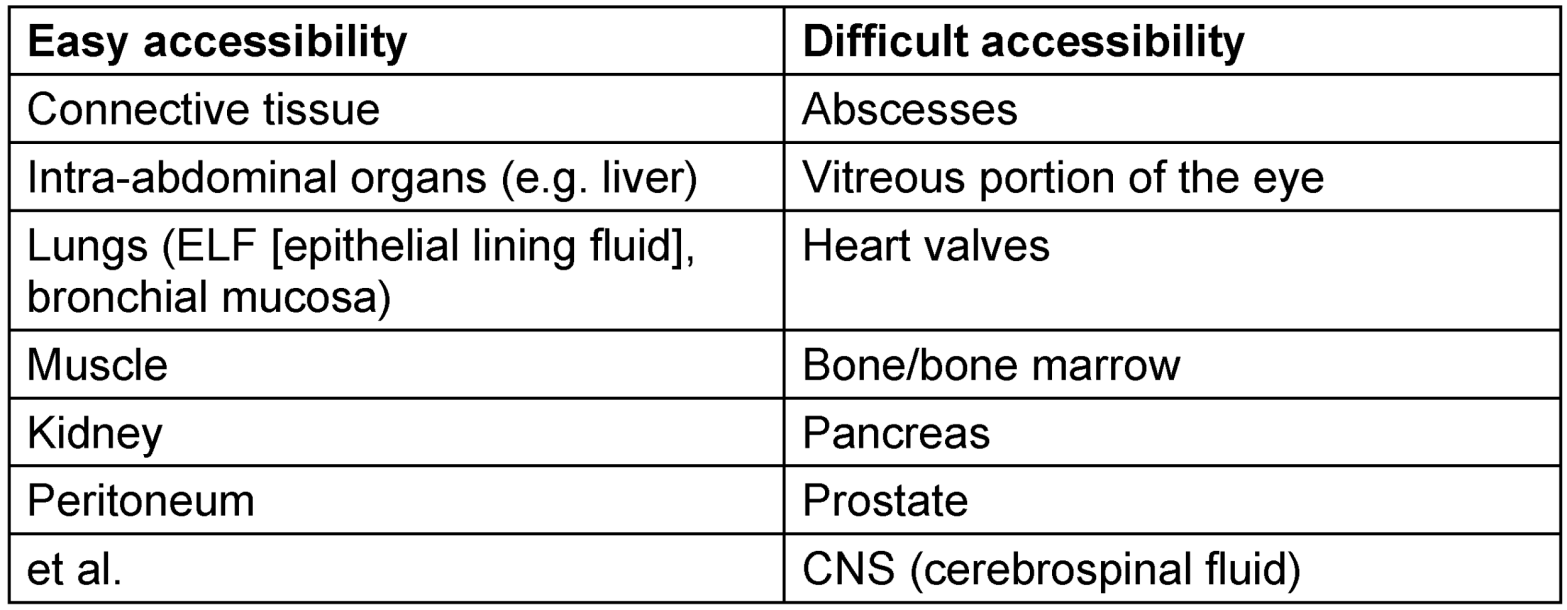
Compartments with easy and difficult access for antibiotics

**Table 3:**
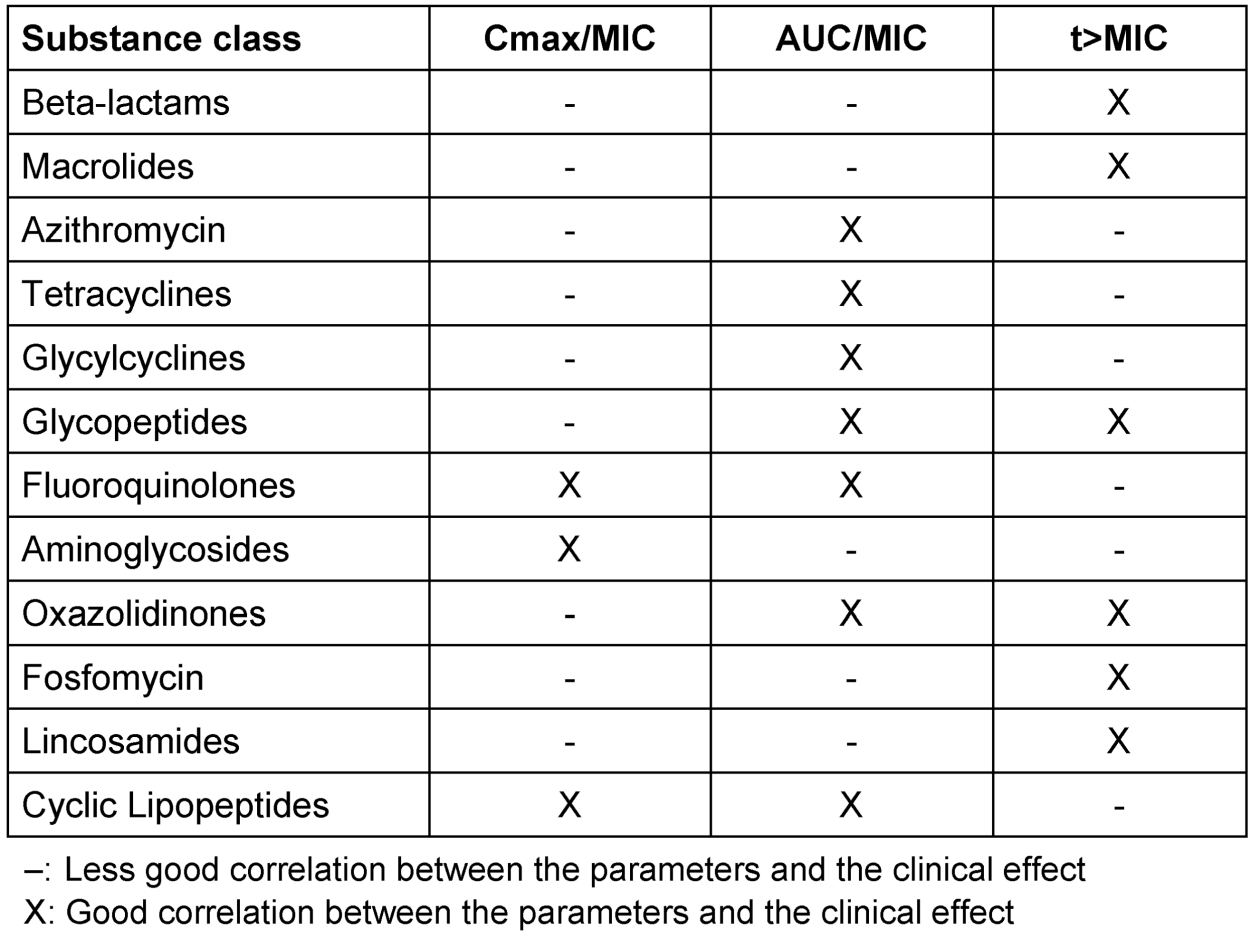
PK/PD parameters of antibiotic groups

**Table 4:**
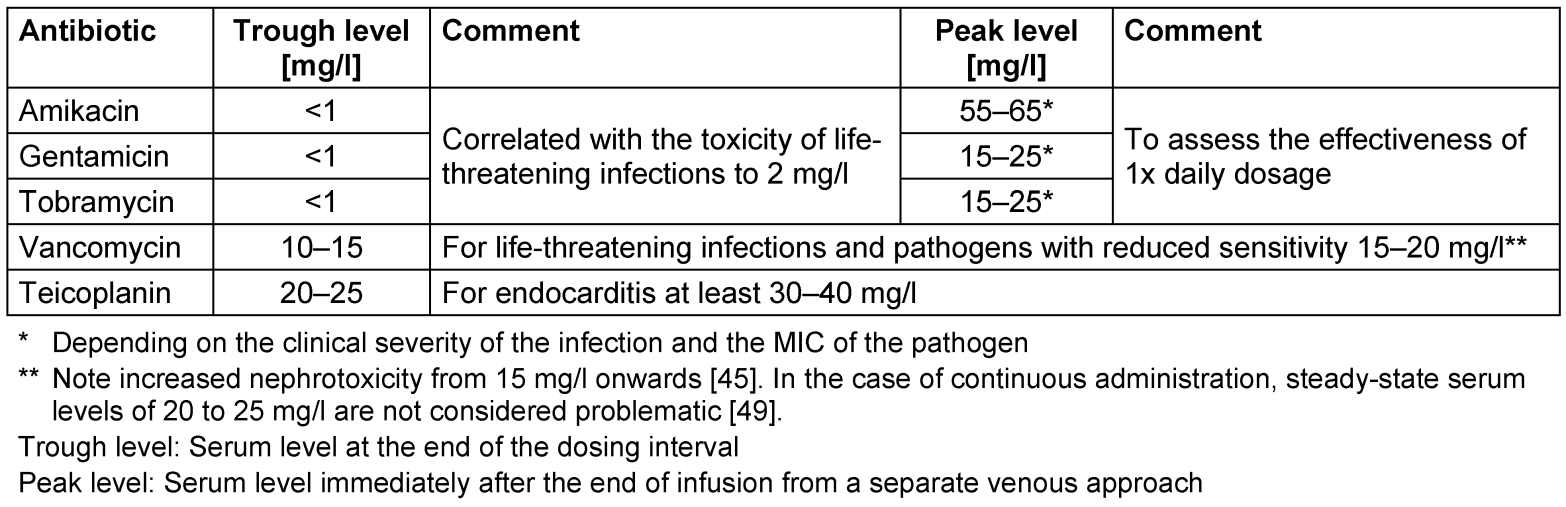
Recommended target ranges for peak and trough levels in the TDM of aminoglycoside and glycopeptide antibiotics (modified according to Burton et al. [15])

**Table 5:**
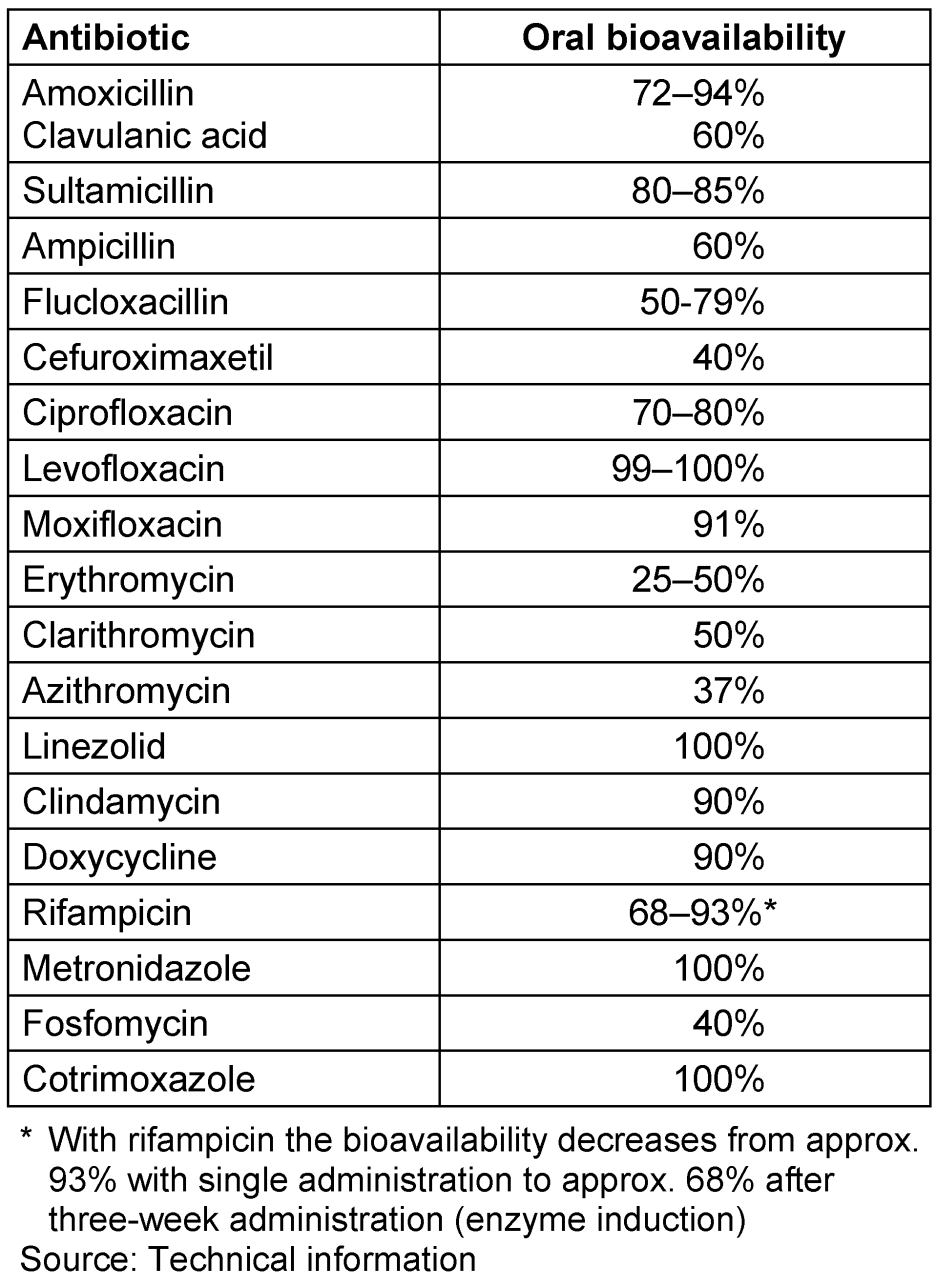
Oral bioavailability of antibiotics for sequential therapy

**Table 6:**
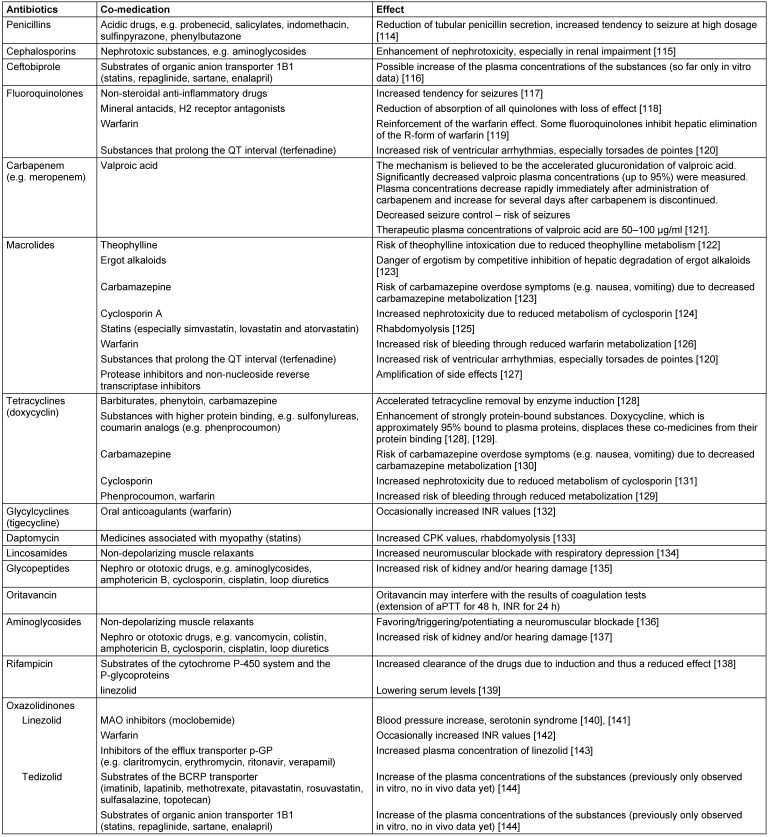
Interactions of antibiotics with other medicines and their consequences
